# G6pd-Deficient Mice Are Protected From Experimental Cerebral Malaria and Liver Injury by Suppressing Proinflammatory Response in the Early Stage of *Plasmodium berghei* Infection

**DOI:** 10.3389/fimmu.2021.719189

**Published:** 2021-08-11

**Authors:** Haoan Yi, Weiyang Jiang, Fang Yang, Fan Li, Yirong Li, Wenjing Zhu, Qing Li, Syed Hassam Fakhar, Yaming Cao, Lan Luo, Wen Zhang, Yongshu He

**Affiliations:** ^1^Department of Cell Biology and Medical Genetics, Kunming Medical University, Kunming, China; ^2^Department of Pathology and Pathophysiology, Kunming Medical University, Kunming, China; ^3^School of Pharmaceutical Sciences and Yunnan Key Laboratory of Pharmacology for Natural Products, Kunming Medical University, Kunming, China; ^4^Department of Human Anatomy/Histology and Embryology, School of Basic Medicine, Kunming Medical University, Kunming, China; ^5^School of Public Health, Kunming Medical University, Kunming, China; ^6^Department of Immunology, College of Basic Medical Sciences, China Medical University, Shenyang, China

**Keywords:** G6PD deficiency, *Plasmodium berghei*, proinflammatory response, experimental cerebral malaria, acute liver injury

## Abstract

Epidemiological studies provide compelling evidence that glucose-6-phosphate dehydrogenase (G6PD) deficiency individuals are relatively protected against *Plasmodium* parasite infection. However, the animal model studies on this subject are lacking. Plus, the underlying mechanism *in vivo* is poorly known. In this study, we used a G6pd-deficient mice infected with the rodent parasite *Plasmodium berghei* (*P.berghei*) to set up a malaria model in mice. We analyzed the pathological progression of experimental cerebral malaria (ECM) and acute liver injury in mice with different G6pd activity infected with *P.berghei*. We performed dual RNA-seq for host-parasite transcriptomics and validated the changes of proinflammatory response in the murine model. G6pd-deficient mice exhibited a survival advantage, less severe ECM and mild liver injury compared to the wild type mice. Analysis based on dual RNA-seq suggests that G6pd-deficient mice are protected from ECM and acute liver injury were related to proinflammatory responses. Th1 differentiation and dendritic cell maturation in the liver and spleen were inhibited in G6pd-deficient mice. The levels of proinflammatory cytokines were reduced, chemokines and vascular adhesion molecules in the brain were significantly down-regulated, these led to decreased cerebral microvascular obstruction in G6pd-deficient mice. We generated the result that G6pd-deficiency mediated protection against ECM and acute liver injury were driven by the regulatory proinflammatory responses. Furthermore, bioinformatics analyses showed that *P.berghei* might occur ribosome loss in G6pd-deficient mice. Our findings provide a novel perspective of the underlying mechanism of G6PD deficiency mediated protection against malaria *in vivo*.

## Introduction

Despite taking increased control measures and prevention, malaria remains a predominant public health problem across the world. *Plasmodium* is the parasite that causes malaria and infects and proliferates in red blood cells (RBCs). The *Plasmodium falciparum* (*P.falciparum*) infection may result in serious complications such as cerebral malaria (CM) (1) and tissue injury (2, 3). During the past, Malaria has been found as a major reason of early mortality around the world subsequently it is explained as sturdiest force for selective pressure on the human genome (4) and is linked with the occurrence of a number of genetic diseases (5, 6). According to a molecular epidemiology survey, glucose-6-phosphate dehydrogenase (G6PD) deficiency has an anti-malaria effect and we found that the G6PD Mahidol 487G>A variant protects against uncomplicated *Plasmodium vivax* (*P.vivax*) infection and reduces disease severity in previous study (6). Furthermore, a large case-control study in Africa has shown that G6PD deficiency protects against CM, but it is associated with increased risk of severe malarial anemia, this study considered the balancing selection hypothesis and indicated that G6PD polymorphism is an evolutionary trade-off of *P.falciparum* infection (7). In previous studies, it was suggested an underlying mechanism for the G6PD deficiency-mediated protection against malaria and found that G6PD-deficient monocytes intensify the phagocytosis of ring-parasitized variant RBCs due to enhanced oxidative stress (8, 9). But current findings lack a description of the process of G6PD deficiency prevents systemic complications. Of note, under normal physiological conditions, G6PD is widely expressed in RBCs as well as in other cells and tissues. Nonetheless, the underlying mechanism of G6PD deficiency-mediated anti-malaria complications effect is not completely delineated.

CM is a known cause of a severe neuropathological complication of *P.falciparum* infection. CD8^+^T cells are well known to play a critical role in CM pathogenesis (10). The current theory holds that the overactivation of the immune system, coupled with the secretion of proinflammatory cytokines, such as TNF-α, IFN-γ, IL-1β, IL-6 and IL-12, correlates with CM pathogenesis (11). These proinflammatory cytokines induce brain vascular endothelial cells to express some chemokines and adhesion molecules, like CD36 (12) and ICAM-1 (13, 14). The interaction between the brain vascular endothelium and immune cells *via* specific adhesion molecules leads to the sequestration of blood cells in brain microvessels and induces blood-brain barrier(BBB) injury (10). Among immune cells, CD8^+^T cells’ sequestration is considered as the main feature in the experimental cerebral malaria (ECM) development (15, 16). The devolvement of ECM is related to the contribution of inflammatory chemokines, CD8^+^T cells and adhesion of infected red blood cells to the vasculature (17).

Imaging immunobiology indicated severe acute liver and lung injury in host during *Plasmodium* infection (18). Humans infected with *P.falciparum* or *P*.*vivax* suffer from hepatic dysfunction, which is associated with the rise of liver enzymes levels in the blood (19, 20). This malarial hepatitis occurs during the blood-stage of *Plasmodium* infection rather than the liver-stage of *Plasmodium* infection since the number of traversed or infected hepatocytes are very low. Endovascular hemolysis, followed by accumulation of free heme and reactive oxidants, and excessive immune response are the prime causes of liver injury induced by *Plasmodium (* 21–23).

The mechanism of resistance to malaria in G6PD deficiency conditions is still unclear. G6PD is a critical enzyme in the pentose phosphate pathway (PPP) (24). It plays an essential role in maintaining cellular redox balance by producing NADPH (25). Cellular G6PD activity is critical for T cells’ immune response and the production of inflammatory cytokines (26). In some autoimmune diseases, G6PD activity is likely to regulate T cells differentiation by affecting the reduction equivalent (27). In addition, G6PD deficiency increases the risk of hemolysis under certain stress conditions (28, 29). Heme could induce expression of Hemoxygenase-1 (HO-1) that favors resistance to *Plasmodium* infections, especially in hereditary hemolytic diseases e.g., HbS (30). G6PD deficiency could also lead to the activation of the nuclear factor erythroid 2-related factor 2 (Nrf2)/HO-1 pathway due to hemolytic response (29, 31). This suggests that the underlying mechanism of G6PD deficiency-mediated anti-CM and acute liver injury involve the regulation of T cells’ immune response and intravascular hemolysis. In this study, we explore the changes in immune response and intravascular hemolysis in G6pd-deficient mice during *Plasmodium berghei* (*P.berghei*) infection.

## Materials and Methods

### Mice and Parasites

G6pd-deficient mice (G6pdx^a-mlNeu^) (32) were backcrossed with C57BL/6 mice more than ten times to change the genetic background. All the mice were housed and fed ad libitum in an environmentally controlled room at 22 ± 1°C with a relative humidity of 50 ± 5% under a light cycle of 12h light/12h dark. DNA was extracted from mice tails, and a 269bp fragment of the G6pd gene was amplified with G6pd_F and G6pd_R (**Supplemental Table 1**). The PCR products were analyzed on a 1.5% agarose gel and sequenced by ABI 3730xl DNA Sequencer with primer G6pd_R (**Supplemental Figure 1A**). The mice were divided into five groups based on the results of Sanger sequencing and the sex of the mice: Wild type(Male), Wild type(Female), Heterozygote, Hemizygote and Homozygote.

*Plasmodium berghei* (strain ANKA 2.34), a rodent malaria parasite, was maintained by mechanical serial passage and used for challenge infections. Blood was collected by cardiac puncture from infected donors when the parasitemia reached 5-8% on day 5 to 7 post-infection. Parasitized red blood cells (pRBCs) were washed with sterile saline three times. 10^6^ pRBCs were intravenously injected into the 5 weeks old experimental mice. Giemsa-stained thin blood films from the mice tail blood have been examined to determine parasitemia levels from day 3 post-infection, and the mortality monitoring was processed daily. Besides the survival analysis, other infectious mice were sacrificed on day 7 post-infection. ECM is strictly dependent on the host genetic background so we used littermates to conduct experiments. All animal experiments were performed in compliance with the requirements of the local animal ethics committee of the Kunming Medical University (KMMU2020438).

### Quantitative Determination of G6pd Enzyme Activity

Quantitative determination of G6pd activity in liver and spleen was performed using a commercial kit (Cominbio, Suzhou, China) according to the manufacturer’s instructions. G6pd enzyme activity was determined by the change of NADP^+^ and NADPH concentration. The absorbance of the mixture was measured at 340nm wavelength using a spectrophotometer. Finally, the G6pd enzyme activity was calculated according to standard curve.

### Measurement of Heme and Free Hemoglobin

Blood was drawn by heart puncture and centrifuged. We collected serum to remove contaminating RBC and passed it through a Microcon YM-3 column (Millipore, Merck, Germany) to remove proteins (MW>3kDa). Total heme was quantified in serum and free heme was quantified in this protein-depleted serum using Quantichrome heme assay kit (Bioassay Systems, USA) according to the manufacturer’s instructions. The serum level of free hemoglobin was measured using commercial kits of Enzyme-Linked Immunosorbent Assay (ELISA) (Meimian industrial, Jiangsu, China) in accordance with the manufacturer’s protocol.

### Evaluation of Blood-Brain Barrier (BBB) Integrity

After ECM symptoms started to appear, five mice from each group were selected for the evaluation of BBB integrity. Briefly, 200μl of 2% Evans blue (Sigma, St. Louis, MO, USA) in PBS was injected intravenously into mice. One hour later, the mice were euthanized and perfused with 30ml of PBS. The brains were removed rapidly and homogenized in PBS (1ml PBS/1g brain weight). Isovolumetric trichloroacetic acid (TCA,100% w/c) was added to the homogenate for 24h at 4°C to extract the Evans blue dye, whose OD values were measured using a spectrophotometer at 620 nm.

### Histological Analysis

Five mice from each group were euthanized, the brain and liver tissues were surgically removed, and fixed immediately in 4% paraformaldehyde fixative solution for 24h, and then dehydrated and embedded in paraffin sections. Sectioning was performed on a microtome to get fine sections slides, which were then stained with hematoxylin and eosin (HE) according to standard procedures to examine microvascular obstruction. Furthermore, immune histology stain was performed for Icam-1(1:200, ab171123, Abcam) and Cd36(1:300, ab80080, Abcam) in brain sections to evaluate the expression of cerebral vascular adhesion molecules. The number of positive blood vessels in 20 visual fields were counted under 400x microscope for each section.

### RNA Extraction and Quantitative Real-Time PCR

Total RNA was extracted from the spleen and brain with RNAiso plus (TaKaRa Biotechnology, Beijing, China). The mRNA was reverse transcribed into cDNA using a PrimeScript RT reagent kit (TaKaRa Biotechnology, Beijing, China). Quantitative real-time PCR was performed by using appropriate primers mentioned in **Supplemental Table 1** and SYBR green PCR master mix (Roche, Penzberg, Germany) for 40 cycles each in an ABI Q6 apparatus (Applied Biosystems, CA, USA). The mRNAs expressions were calculated using the ratio of the gene of interest to β-actin. All qRT-PCR assays were performed in triplicate.

### Hematology Analysis and Biochemical Assay

Peripheral blood samples were collected. RBCs count, hemoglobin concentration, mean corpuscular volume (MCV), mean corpuscular hemoglobin (MCH) and mean corpuscular hemoglobin concentration (MCHC) were determined by using an automated blood analyzer (XT-2000i, Sysmex, Japan). Blood without anticoagulants was centrifuged at 4000g for 10 min and the clear supernatants were collected. Alanine aminotransferase (ALT), aspartate transaminase (AST) and alkaline phosphatase (ALP) in serum were measured to assess the biochemical level of hepatocellular injury using an automated biochemistry analyzer (Hitachi 7060, Japan).

### Cytokines Detections

The serum levels of TNF-α, IFN-γ, IL-1β, IL-6, IL-12, TGF-β and IL-10 were measured using commercial enzyme-linked immunosorbent assay (ELISA) kits (Meimian industrial, Jiangsu, China) according to the manufacturer’s protocol. The concentrations of the cytokines were calculated using standard curves which were constructed using serial dilutions of cytokines standards provided within the kit. All cytokines were measured in triplicates.

### Flow Cytometry Analysis

Lymphocytes from the liver, spleen and brain were extracted with a percoll gradient to analyze cells population differentiation. The differentiation of Th1 cells(CD45^+^CD3^+^CD4^+^IFN-γ^+^), Treg cells(CD45^+^CD4^+^CD25^+^Foxp3^+^) and DCs(CD45^+^CD11c^+^CD80^+^CD86^+^MHCII^+^) from the liver and spleen,CD8^+^T cells (CD45^+^CD3^+^CD8^+^Granzyme B^+^) from the brain were detected by flow cytometry. We use BV421-anti-CD45(103134, Biolegend, USA) labeled leukocytes to exclude parenchymal cells interference. To detect the maturation of DCs, cells were stained with FITC-anti-CD11c (117306, Biolegend, USA),PE-anti-CD86 (159204, Biolegend, USA), PERCP_CY5.5-anti-MHCII (107626, Biolegend, USA) and APC-anti-CD80(104714, Biolegend, USA). To detect the activation of Tregs, cells were incubated with PerCP-anti-CD4 (100432, Biolegend, USA) and PE-anti-CD25 (102008, Biolegend, USA) for surface staining. Subsequently, cells were fixed and permeabilized for intracellular staining using APC-anti-Foxp3 (17-5773-82, eBioscience, USA). To detect the differentiation of Th1, 10^6^ fresh lymphocytes were stimulated in 24-well plates with 50 ng/ml phorbol myristate acetate (PMA, Cayman, USA) and 750ng/ml ionomycin (Cayman, USA) for 6 h at 37°C and 5% CO_2_, 10μg/ml Brefeldin A (Beyotime, Beijing, China) was added to block cytokine secretion. The cells were stained with FITC-anti-CD3 (100204, Biolegend, USA) and PerCP-anti-CD4 and PE-anti-IFN-γ (163503, Biolegend, USA) staining was performed after fixation and permeabilization. The lymphocytes from the brain were collected and were stained with FITC-anti-CD3 and PerCP-anti-CD8(100732, Biolegend, USA), APC-anti-granzyme B (17-8898-82, eBioscience, USA) staining was performed after fixation and permeabilization. Representative gating strategy is shown in **Supplemental Figure 2**.

### Western Blot

Total protein was extracted with RIPA lysate added with PMSF from spleen and liver, 50μg of the proteins was loaded in the lane. The proteins were separated by 10% SDS-PAGE gel, transferred to a polyvinylidene fluoride (PVDF) membrane and blocked with 5% milk for 60 min at room temperature. Immunoblot analysis was carried out using anti-Nrf2 monoclonal antibody (1:1000, sc-365949, SANTA), anti-Ho-1 monoclonal antibody (1:2000, ab13248, Abcam) and anti-β-actin monoclonal antibody (1:1000, ab7276, Abcam) for incubating overnight at 4°C. The HRP‐secondary antibody bound to the primary antibody. The developed image was detected using a chemiluminescence detection imaging system. For quantification, Image J software was used to quantify at least three independent experiments for the western blot band. β-actin was used as a loading control to calculate the relative quantification among each group.

### Dual RNA-Seq and Bioinformatic Analysis

Dual RNA-seq (33) was performed to identify the interaction of mice with *P.berghei*. RNA libraries were generated using Illumina’s TruSeq Stranded Total RNA Sample Prep Kit with input amounts of at least 1μg total RNA from the liver. RNA libraries were sequenced using paired-end 150 bp reads on a NovaSeq system (Illumina, San Diego, CA, USA). Raw sequencing files were subjected to quality control using FASTQC. The raw reads data were aligned to the C57/B6J mouse genome (release 100 in Ensemble) by using Hisat2 (34). Unmapped reads were extracted and aligned to the *P.berghei* genome (release 47 in PlasmoDb). Differential expression analysis was performed using DESeq2 (35) and GSEA (Gene Set Enrichment Analysis) on transcriptome changes was also performed using the KEGG (Kyoto Encyclopedia of Genes and Genomes) database. BiNGO was used to enrich GO(Gene Ontology) categories with differentially expressed genes (36). The PPI (Protein-Protein Interaction) network was predicted using STRING online database (http://string-db.org). Cytoscape 3.8.0 was used to construct the network and cytoHubba module was used to find the key nodes.

### Statistical Analysis

Data were presented as means and standard error (Mean ± SEM). Differences were compared using a one-way ANOVA test and multiple comparisons using SNK-Q test. Survival analysis was performed using the Kaplan-Meier log-rank test. Statistical analyses were performed using the SPSS 21.0 and GraphPad Prism 8.0 statistical software. The statistical significance level was set at α = 0.05.

## Results

### G6pd Deficiency Enhances Survival and Alleviates Liver Injury in Mice During *Plasmodium berghei* Infection

To determine whether G6pd gene mutation affects the enzyme activity in tissues, we measured G6pd activity in the liver and spleen of different genotype mice, including the wild type (Male), wild type (Female), heterozygote, hemizygote and homozygote. As expected, the enzyme activity was the highest in the wild type mice groups while the hemizygote and homozygote mice displayed the lowest enzyme activity (P<0.0001, One-way ANOVA and SNK-Q test, **Supplemental Figure 1B**). The data showed that there is statistically significant difference between the enzyme activity level of the wild type mice and the G6pd-deficient mice.

**Figure 1:**
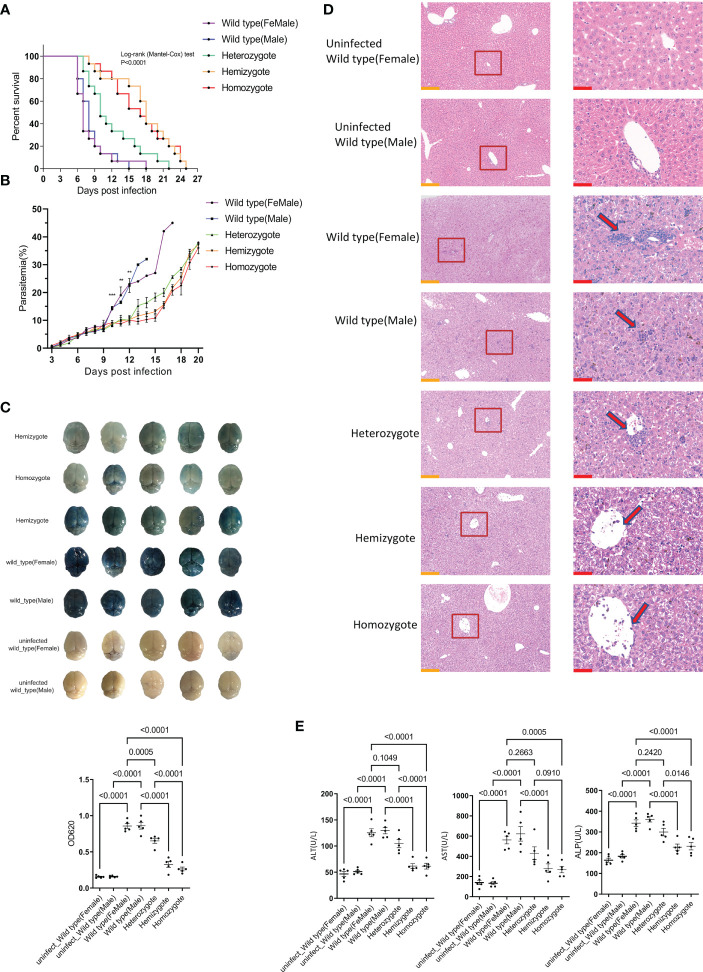
G6pd-deficient mice infected with *P.berghei* showed milder complications compared to wild type mice. **(A)** Survival analysis of wild type and G6pd-deficient mice infected with *P.berghe*i. Mice were injected intraperitoneally with 10^6^ pRBCs infected with *P.berghei*, infected mice were monitored daily for survival. (n =15/each group, data are combined from three independent experiments) **(B)** Dynamics change of parasitemia. Parasitemia was monitored until the mice died or day 20 post-infection. **(C)** Whole brains of Evans blue dye leakage in wild type and G6pd-deficient mice and quantification analysis of Evans blue dye extravasation in the brain using spectrophotometer. (n = 5/each group, data are representative of three independent experiments) **(D)** Representative images of HE staining of the liver showed that the level of immune cell infiltration (arrows) was reduced in G6pd-deficient mice infection with *P.berghei* on day 7 post-infection. The contents present in the red box are indicative of the high magnification observation of the display. Yellow scale bars, 200μm; Red scale bars,50μm. (n = 5/each group, data are representative of three independent experiments) **(E)** Serum enzymatic indicators showed that G6pd-deficient mice had less liver injury in response to *P.berghei* infection compared to wild type mice on day 7 post-infection. (n = 5/each group, data are representative of three independent experiments).

Given that G6PD deficiency favors malaria resistance, we investigated whether it plays a role in the ECM pathological process during the *P.berghei* infection. Fifteen mice of each group were given an intraperitoneal injection of 10^6^ pRBCs infected with *P.berghei*. Daily monitoring revealed significant survival difference between G6pd-deficient mice and wild type mice (P<0.0001, Kaplan-Meier analysis, **Figure 1A**). In wild type group, more than 80% of the mice died between 5 to 9 days post-infection exhibiting typical ECM symptoms, such as drop in body temperature and neurological manifestations (e.g., ataxia and paralysis). In contrast, in G6pd deficiency group, only less than 20% (heterozygote) and 40% (hemizygote and homozygote) of the mice died during this period, whereas rest of the mice died between 12 to 24 days post-infection and mostly died from anemia.

Next, we measured the parasitemia in each group of survived mice from day 3 post-infection. The results showed no statistically significant difference in parasitemia before day 9 post-infection. However, a significantly low parasitemia level was associated with G6pd deficiency after day 9 post-infection (P<0.05, One-way ANOVA test, **Figure 1B**). The occurrence of CM is closely related to the integrity of BBB. On day 7 post-infection, five mice from each group were selected for the evaluation of BBB integrity. We found that the brains from G6pd-deficient mice appeared visually milder blue than those from wild type mice, which appeared darker blue. As expected, the OD_620_ of Evans blue from G6pd-deficient mice brains were significantly lower than wild type mice. The less the G6pd activity was, the less extensive was BBB leakage (P<0.0001, One-way ANOVA and SNK-Q test, **Figure 1C**). These results suggest that G6pd deficiency protects the BBB during *P.berghei* infection.

On day 7 post-infection, five mice from each group were selected for the evaluation of liver injury. The histologic examination of livers by HE staining revealed more lymphocyte infiltration in the wild type mice than the G6pd-deficient mice during *P.berghei* infection (**Figure 1D**). Further, we analyzed ALT, AST and ALP in the serum of mice from each group and found that all the three enzymes’ activities were lower in G6pd-deficient mice than in wild type mice, suggesting moderate liver injury in the G6pd-deficient mice (P<0.0001, One-way ANOVA and SNK-Q test, **Figure 1E**). These results shows that G6pd deficiency suppresses immune cell infiltration and reduces liver injury.

### G6pd Deficiency Does Not Induce Hemolysis in the Early Stages of *Plasmodium berghei* Infection

The *Plasmodium* infects erythrocytes, resulting in their dysfunction, severe hemolysis and intravascular heme release, and leading to tissue injury and resist to ECM. To investigate the hemolysis level in G6pd-deficient mice at the early stages of *P.berghei* infection, we measured the hematological parameters including RBC count, Hb, MCV, MCH. We found that there was no significant difference among groups on post-infection day 7 (P>0.05, One-way ANOVA test, **Supplemental Table 2**). Furthermore, we found malaria infection induces total heme, free heme and free hemoglobin elevation but no significant difference among *P.berghei* infected groups(P<0.05,One-way ANOVA test, **Supplemental Figures 3A, B**). HO-1 is a cytoprotective enzyme that transforms free heme into carbon monoxide, iron and biliverdin to maintain cellular redox homeostasis and its expression can be induced by heme floating in serum. Our results indicate that malaria infection induces Ho-1 elevation in serum, but G6pd deficiency does not reduce Ho-1 elevation in serum (P<0.05, One-way ANOVA test, **Supplemental Figure 3C**). Nrf2/Ho-1 pathway provides protection against severe malaria. To clarify whether G6pd deficiency modulates Nrf2 and Ho-1 proteins expressions in the early stages of *P.berghei* infection, we examined and quantified the Nrf2 and Ho-1 proteins expressions in mice liver and spleen on day 7 post-infection. The western blot analysis showed that the protein expression levels of Nrf2 and Ho-1 were significantly up-regulated after *P.berghei* infection but did not change significantly in infected mice (P<0.05, One-way ANOVA test, **Supplemental Figure 3D**), suggesting that G6pd deficiency did not affect the Nrf2/Ho-1 pathway in the early stages of *P.berghei* infection. These results demonstrate that the G6pd deficiency mediated resistance mechanism in CM is likely independent of hemolysis and Nrf2/Ho-1 pathway at the early stage of *P.berghei* infection.

### Transcriptome Analysis Showed That G6pd Deficiency Suppress Proinflammatory Response

To further understand the relation between *P.berghei* infection and G6pd deficiency, RNA-seq analysis was performed on ten infected mice livers (five wild type males and five hemizygotes, at least 10Gb data) and ten uninfected mice livers (five wild type males and five hemizygotes, at least 6Gb data). The read counts had been normalized using DESeq2 before the analysis. The tSNE cluster analysis indicated that *P.berghei* infection and G6pd deficiency are independent factors of mouse transcriptome variation. We also found that *Plasmodium* has different gene expression variation in G6pd-deficient mice and wild type mice (**Supplemental Figure 4**).

**Figure 2:**
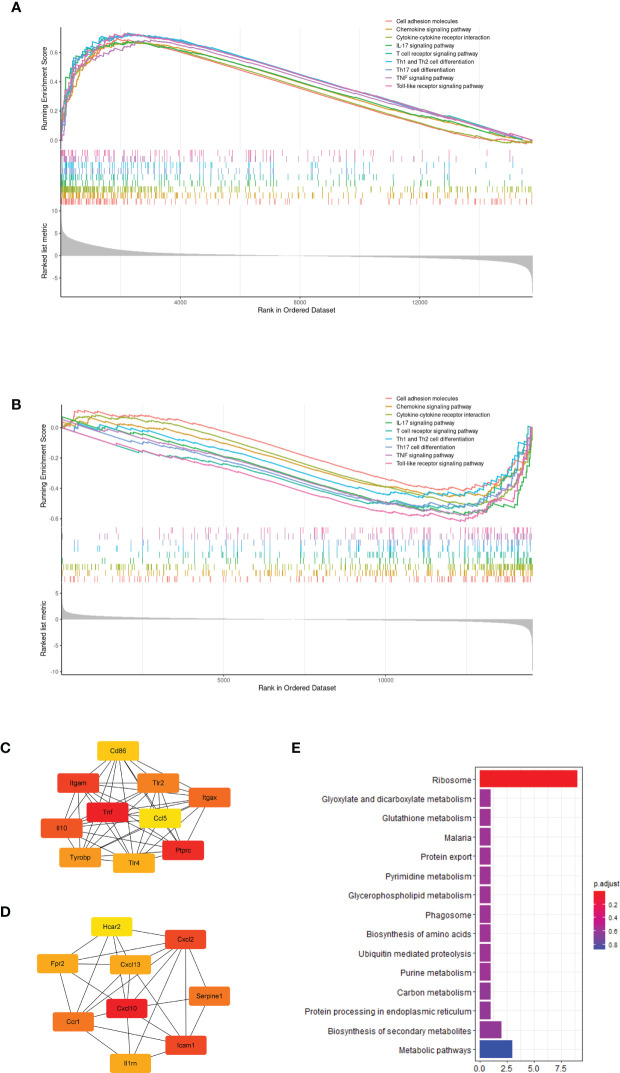
Bioinformatics analysis based on transcriptome showed that G6pd-deficient mice altered the immune response in response to *P.berghei* infection. **(A)** GSEA showed that *P.berghei* infected wild type mice significantly upregulated the immune response compared to uninfected mice on day 7 post-infection. **(B)** GSEA showed that G6pd-deficient mice down-regulated immune response compared to wild type mice on day 7 post-infection. **(C)** PPI analysis based on significantly differentially expressed genes(uninfected wild mice compared to infected wild mice) showed that *P.berghei* infection induced host antigen recognition and immune response compared to uninfected mice on day 7 post-infection. The redder the color, the more critical the gene is in the network. **(D)** PPI analysis based on significantly differentially expressed genes(infected wild mice compared to infected hemizygous mice) showed that G6pd-deficient mice suppressed the immune response induced by *P.berghei* infection compared to wild type mice on day 7 post-infection. The redder the color, the more critical the gene is in the network. **(E)** KEGG enrichment of differentially expressed genes of *Plasmodium* parasites on different hosts. Unmapped reads were extracted and aligned to the *P.berghei* genome and differentially expressed genes were identified and enriched with KEGG database. The ribosome loss maybe occurs in *P.berghe*i parasites on the G6pd-deficient mice.

Further, we performed GSEA to evaluate transcriptome changes based on the KEGG database. First, we compared the infected and uninfected wild type mice. The GSEA results showed that most immune related pathways, including innate immune pathway, Th1 and Th17 cells differentiation pathway, chemokine interactions and cell adhesion were significantly up-regulated after *P.berghei* infection (**Figure 2A**). Next, we analyzed the effect of G6pd deficiency in *P.berghei* infection. GSEA enrichment results showed that G6pd deficiency mainly down-regulates innate immune responses, adaptive immune responses, chemokines interactions and cell adhesion related pathways (**Figure 2B**). These results suggest that immune related response involved G6pd deficiency mediated protection to *P.berghei* infection. Moreover, we identified the differentially expressed genes through rigorous statistical comparisons (log_2_foldchange>1.2 and adjusted p-value<0.05). A total of 1759 differentially expressed genes, including 1324 up-regulated and 435 down-regulated expressed genes were identified by comparison of the *P.berghei* infected wild group with the uninfected wild type mice. A total of 574 differentially expressed genes, including 277 up-regulated genes and 297 down-regulated expressed genes were identified by comparison of the *P.berghei* infected hemizygote group with the *P.berghei* infected wild group. PPI network analysis was conducted using the differentially expressed genes based on STRING database. Cytoscape was used to map the network and cytoHubba module were used to find the key nodes. The results showed that the critical network after *P.berghei* infection is related to immune response (**Figure 2C**). Additionally, the critical network of *P.berghei* infection in G6pd-deficient mice were also closely related to proinflammatory response (**Figure 2D**). To further demonstrate that proinflammatory responses play an important role in this process, we enriched the differentially expressed genes with the GO database. The results showed that most of the TOP20 significant pathways were linked with the immune response and proinflammatory response (**Supplemental Table 3**). It is worth noticing that the complications of malaria were closely linked to excessive proinflammatory response. These bioinformatics results suggest that G6pd deficiency could protect against excessive immune responses, caused by *P.berghei* infection, and thus suppress malaria complications.

Moreover, we extracted the transcriptome of *P.berghei* from dual RNA-seq raw reads and found that most of the ribosomal related genes of *P.berghei* were significantly down-regulated in G6pd-deficient mice, suggesting that ribosomal loss may occurs in *P.berghei* parasites on G6pd-deficient mice (**Figure 2E**).

### G6pd Deficiency Affects the Differentiation of T Cells and Maturation of Dendritic Cells in *P.berghei* Infected Mice

**Figure 3:**
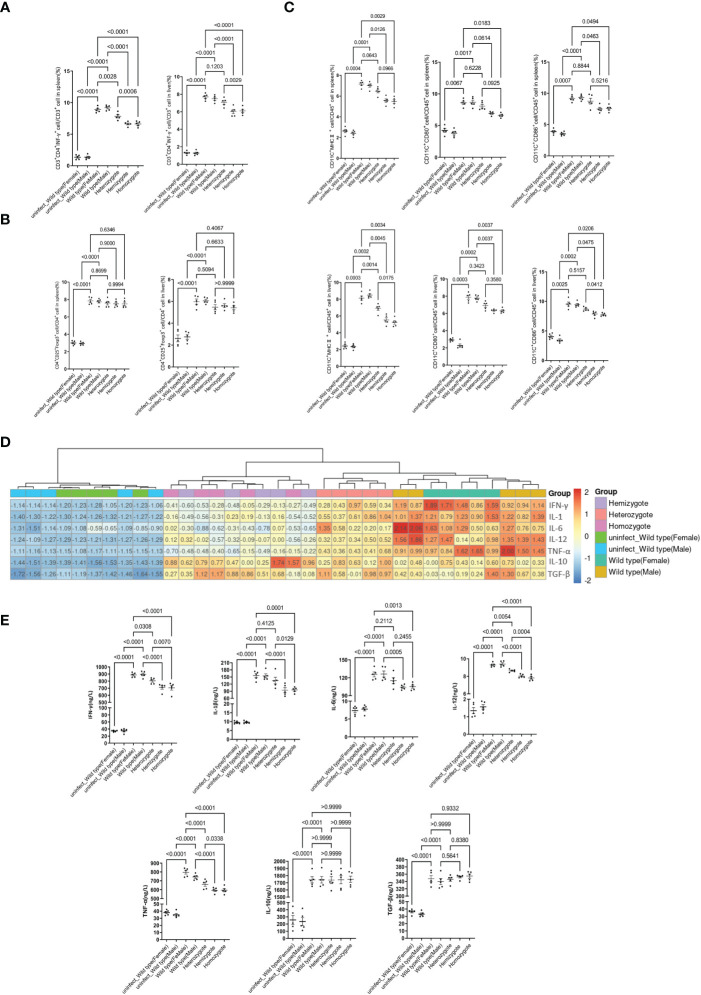
G6pd-deficient mice suppresses the proinflammatory response after *P.berghei* infection. **(A)** Frequencies of the respective Th1 populations in the spleen and liver on day 7 post-infection. **(B)** Frequencies of the respective Treg populations in the spleen and liver on day 7 post-infection. **(C)** Frequencies of the mature DCs in the spleen and liver on day 7 post-infection. **(D)** Heatmap of relative cytokine gene expression in the spleens on day 7 post-infection. Expression levels were quantified by real-time quantitative PCR with β-actin as the internal reference and normalized. **(E)** Serum IFN-γ, IL-1β, IL-6, IL-12, TNF-α, IL-10 and TGF-β levels were quantified by ELISA on day 7 post-infection. (n = 5/each group, data are representative of three independent experiments).

To further determine whether G6pd deficiency suppresses proinflammatory response, alleviates liver injury and prevents ECM, we extracted lymphocytes from the liver and spleen of mice from each group to detect the differentiation of Th1 cells (CD45^+^CD3^+^CD4^+^IFN-γ^+^) and Treg cells (CD45^+^CD4^+^CD25^+^Foxp3^+^) by flow cytometry. The results showed that the differentiation of Th1 cells gradually decreased with the reduction of G6pd enzyme activity after *P.berghei* infection(P<0.0001, One-way ANOVA and SNK-Q test, **Figure 3A**). Meanwhile, the differentiation of Treg cells gradually increased after *P.berghei* infection but no statistical difference among *P.berghei* infected groups (P<0.05, One-way ANOVA test, **Figure 3B**). These results indicate that G6PD deficiency inhibits the differentiation of Th1 in *P.berghei* infected mice.

The maturation of DCs(CD45^+^CD11c^+^CD80^+^CD86^+^MHCII^+^) also plays an important role in the occurrence of CM, therefore we examined the maturation status of DCs. We selected CD80, CD86 and MHCII as markers for DCs mature and the results displayed significantly lower mature rate of DC in G6pd-deficient mice as compared to wild type mice (P<0.0001, One-way ANOVA and SNK-Q test, **Figure 3C**). These results indicate that G6pd deficiency inhibits the mature rate of DCs in *P.berghei* infected mice.

### G6pd Deficiency Down-Regulates the Expression of Proinflammatory Cytokines

The malaria pathogenesis development involves inflammatory processes. ECM is closely related to cytokines *in vivo*. To further understand the role of cytokines in the context of G6pd deficiency, we measured the expression of *TNF-α*, *IFN-γ*, *IL-1β*, *IL-6*, *IL-12*, *TGF-β* and *IL-10* along with cytokine levels in serum and mRNA expression in spleen. The results showed that the expression levels of proinflammatory cytokines *TNF-α, IFN-γ, IL-1β, IL-6* and *IL-12* decreased significantly, but there was no statistically significant difference for the cytokines levels of *TGF-β* and *IL-10* (One-way ANOVA test, **Figures 3D, E**).

### G6pd Deficiency Reduces the Expression of Chemokines and Adhesion Molecules

**Figure 4:**
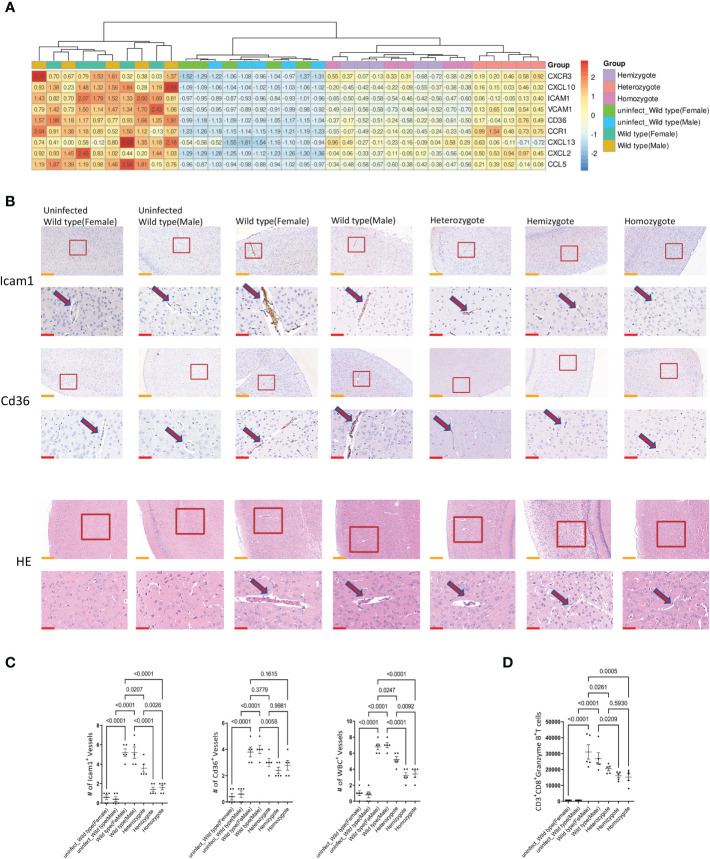
G6pd-deficient mice reduced chemokines and adhesion molecules expression in brain compared with wild type mice in response to *P.berghei* infection. **(A)** Heatmap of relative chemokines and adhesion molecules expression in brain on day 7 post-infection. Expression levels were quantified by real-time quantitative PCR with β-actin as the internal reference and normalized. **(B)** Brain pathology of mice. Representative images of brain sections with the microvessels (arrows) from uninfected and *P.berghei* infected mice on day 7 post-infection. The contents present in the red box are indicative of the high magnification observation of the display. Yellow scale bars, 200μm; Red scale bars,50μm. **(C)** The plot graphs show quantification of the data in **(B)**. Microvessels per microscopic field were quantified in 20 fields per mouse and the values are presented in the corresponding plot graphs. **(D)** The cell number of CD8^+^T cells (CD45^+^CD3^+^CD8^+^Granzyme B^+^) in brain were quantified. (n = 5/each group, data are representative of three independent experiments.).

Chemokines play key roles in the generation and delivery of immune and inflammatory responses. ECM is associated with increased expression of brain chemokines and adhesion molecules due to proinflammatory cytokines *in vivo*. In this study, we investigated the genes expressions of chemokines and adhesion molecules (*Cxcr3, Cxcl10, Icam1, Vcam1, Cd36, Ccr1, Cxcl13, Cxcl*2 and *Ccl5*) in the brain of mice. The results of quantitative real-time PCR showed that G6pd deficiency significantly reduced the mRNA expression of chemokines and adhesion molecules in the brain (P<0.0001, One-way ANOVA and SNK-Q test, **Figure 4A**). Additionally, we used immunohistochemistry to perform a semi-quantitative analysis of Icam1 and Cd36 in brain. The results showed that G6pd deficiency significantly reduced the positive blood vessel levels of Icam1 and Cd36 (P<0.0001, One-way ANOVA and SNK-Q test, **Figures 4B, C**).

### G6pd Deficiency Alleviates the Obstruction of Brain Microvessels

Microvascular obstruction caused by the overexpression of chemokines and adhesion molecules in blood vessels is the main cause of ECM. We observed the cerebral microvascular obstruction by HE staining and found that the number of cerebral microvascular obstruction is low in G6pd-deficient mice during the *Plasmodium* infection. In addition, the number of blocked microvessels was also significantly lower (P<0.0001, One-way ANOVA and SNK-Q test, **Figures 4B, C**). CD8^+^T cells are the principal source of microvascular obstruction during ECM. Subsequently, we used flow cytometry to observe the accumulation of CD8^+^T cells (CD45^+^CD3^+^CD8^+^Granzyme B^+^) in the brain. The results showed that the absolute number of CD8^+^T cells were significantly lower in G6pd-deficient mice (P<0.0001, One-way ANOVA and SNK-Q test, **Figure 4D**). These results suggest that the G6pd deficiency leads to the remission of cerebral microvascular obstruction and prevents the occurrence of ECM.

## Discussion

T-cell mediated proinflammatory response plays a crucial role in malaria and its subsets trigger host resistance to *Plasmodium* infection (37). Both CM and malaria induced liver injury are associated with excessive immunity response and proinflammatory cytokines’ secretion (38). Cytokines, chemokines and vascular adhesion molecules cause leukocytes accumulation in the brain which obstruct brain microvessels that leads to the damage of brain endothelial cells (11). Liver immanent immune system made up of specialized liver cell populations participate in the host defense against the malaria parasite. Nevertheless, Liver immune cells infiltration was favored by cytokines and chemokines and even in the absence of liver-stage *Plasmodium*, it could cause liver injury (22). Thus, the excessive immune response protects against *Plasmodium* infection, but induces the development of a series of complications.

G6PD deficiency, which has long been thought to be a natural selection product of malaria, as evidenced by numerous molecular epidemiological studies, is an X-linked inherited disease and is one of the most common enzymopathies (24). Recent studies have shown that a novel non-steroidal G6PD inhibitor blocks T cell-produced inflammatory cytokines IFN-γ, TNF-α, IL-2 and TNF-α *in vitro*, and suggested that G6PD is a pharmacological target for modulating immune response (26). The complications of malaria infection are mostly related to excessive proinflammatory response. However, it is unclear whether G6PD deficiency can alleviate the CM and acute liver injury complications *via* suppressing the immune response. In this study, we investigated this question by infecting Gp6d-deficient mice with *P.berghei*.

We first validated the resistance of G6pd-deficient mice to *P.berghei* infection. We evaluated the survival time of mice, the integrity of BBB, lymphocyte infiltration in liver, and the activities of ALT, AST and ALP in serum. The data revealed that G6pd-deficient mice infected with *P.berghei* showed extended survival, enhanced blood-brain barrier integrity and decreased levels of liver injury markers. HE staining of the mice liver tissues after *P.berghei* infection demonstrated that the degree of immune cells infiltration was significantly lower in G6pd deficiency context. Taken together, the results demonstrated that G6pd-deficient mice infected with *P.berghei* showed markedly moderate ECM symptoms and liver injury compared to the wild type mice.

G6PD deficiency can cause hemolysis, triggered by infections or certain drugs, which may be life-threatening (39). It is unclear whether G6pd deficiency causes more severe hemolysis at early stage in malaria. We counted the blood cells and measured the heme and free hemoglobin in mice infected by *P.berghei*. We found that the level of hemolysis was not significantly different between G6pd-deficient mice and wild type mice on post-infection day 7. Ho-1 expression can be significantly induced by heme that mediates the enzymatic cleavage of heme. Nrf2/Ho-1 pathway plays an important role in the resistance to malaria and the occurrence of ECM, Ho-1 was thought to prevent the ECM development and liver failure (40, 41). In the meantime, Nrf2/HO-1 pathway is the main defense strategy for hemolysis. G6PD deficiency may lead to hemolysis and the event is closely related to the Nrf2/HO-1pathway. In the present study, we found that the protein expression level of Nrf2 and Ho-1 were not significantly changed, indicating that G6pd deficiency mediated malaria resistance is independent of the Nrf2/Ho-1 pathway in the early stage of *P.berghei* infection. This also suggests that hemolysis is not a factor in the resistance to ECM development in G6pd-deficient mice in the early stage of *P.berghei* infection.

To understand the interaction between G6pd deficiency and *P.berghei* infection, we conducted dual RNA-seq for G6pd-deficient mice and *P.berghei* pathogen on day 7 post-infection. Using dual RNA-seq in liver tissue, we can obtain and analyze the transcriptome information of kinetic gene expression in both G6pd-deficient mice and *P.berghei* during infection. During the analysis of the host mice data, we found that the gene expression changes in the transcriptome, caused by *P.berghei* infection and G6pd deficiency, was to change immune response mainly. The PPI analysis based on the differentially expressed genes showed that the most critical network were antigen recognition and proinflammatory response during *P.berghei* infection while G6pd deficiency reduces the immune response and the gene expression level of corresponding chemokines and cytokines. Changes in immune responses and proinflammatory responses were also found in GO enrichment. These results suggest that the main change in G6pd-deficient mice during *Plasmodium* infection is the alteration of the host’s immune response. This study provides the first *P.berghei* transcriptome data in the context of G6pd deficiency and indicates its unique genes expression changes in G6pd deficiency.

Above bioinformatics analysis showed that G6pd deficiency prevents the host’s immune response during malaria. The excessive immune response is a critical factor that is linked to complications associated with malaria. Excessive Th1 responses mediated by Th1 cells and their related inflammatory cytokines promote ECM (42). Treg proliferation might be associated with the suppression of immoderate proinflammatory Th1 response during early malaria infection (43). Malaria-induced liver injury is also due to excessive pro-inflammatory cell infiltration (22). Our study showed a significantly less differentiation of Th1 cells in G6pd-deficient mice with malaria infection in liver and spleen. Treg, however, showed no statistically significant difference. Maturation of DCs is essential to the proinflammatory response induced by *Plasmodium.* We selected CD80, CD86 and MHCII as markers for maturation of DCs and also used to detect maturation of DCs (44). Our results showed that the maturation of DCs were significantly reduced in liver and spleen of mice with G6pd deficiency. In this study, the differentiation and maturation of Th1 and DCs was limited in the liver and spleen of G6pd-deficient mice infected with *P.berghei*, meaning that the excessive immune response be suppressed.

A balanced proinflammatory and regulatory cytokines play a role during malaria infection (45). Among the proinflammatory cytokines, the levels of TNF-α (46) and IFN-γ (47) were significantly associated with death from ECM. Cyclic paroxysms and high fever are hallmarks of malaria and are associated with high levels of IL-1β, one of the pyrogenic cytokines (48). TGF-β and IL-6 have a regulatory effect on the differentiation of DCs, Treg and Th17 (49). IL-12 can alter TNF-α and IFN-γ expressions during *Plasmodium* infection (50). To determine whether G6pd deficiency can affect cytokines production during malaria infection, we first examined the transcription level in the spleen. We found that the gene transcription level of proinflammatory cytokines was significantly down-regulated in the spleen of G6pd-deficient mice infected with *P.berghei*. Further, we used ELISA to detect cytokines levels in the serum. We also found that the levels of proinflammatory cytokines in the serum of G6pd-deficient mice were significantly lower. These results were consistent with those obtained by flow cytometry and transcriptome analyses.G6pd deficiency suppresses the expression of pro-inflammatory cytokines that further suppress malaria-related complications which protect organs such as the liver and brain from immune damage.

Cerebral chemokines and adhesion molecules induced by proinflammatory cytokines in the serum play an important role in the process of ECM (51, 52). Therefore, we quantified and analyzed the transcription level of brain chemokines and adhesion molecules and found that chemokines and adhesion molecules related gene transcription in G6pd-deficient mice infected with *P.berghei* was significantly lower than the wild type mice. Further,we found that the positive vascular rate of adhesion molecules (Cd36 and Icam1) expression was lower in G6pd-deficient mice. At the same time, HE staining also showed the cutback of cerebral microvascular obstruction in G6pd-deficient mice with *Plasmodium* infection. Overexpressed chemokines and adhesion molecules in the brain recruit CD8^+^T cells which through the cytotoxic effector molecule granzyme B, causes injury to the brain’s blood-brain barrier as well as ECM symptoms (53, 54). We discovered a significantly lower number of CD45^+^CD3^+^CD8^+^Granzyme B^+^T cells in the brains of G6pd-deficient mice compared to the wild type mice. Plus, the expressions of chemokines were lower in the G6pd-deficient mice. These results suggest that G6pd deficiency reduces the proinflammatory response during malaria by suppressing the overactive immune response in the body, leading to a diminished cerebral microvascular obstruction and subsequently a reduction of the incidence and severity of ECM.

Our transcriptome data from dual RNA-seq raw reads showed that ribosome protein genes exhibited low expression, and ribosome loss might occur in *P.berghei* parasites on the G6pd-deficient mice. Ribosome plays a critical role in protein synthesis and protein homeostasis of living organisms. Ribosomal function is associated with longevity and protein homeostasis. The result was consonant with the data of the decreasing parasitemia in G6pd-deficient mice. We speculate that the ribosome loss alters the parasite’s protein synthesis function and lead to a change of protein homeostasis and disruption of the parasite survival, further tests are expected to affirm the outcomes.

In summary, our results suggest that G6pd deficiency impedes the ECM caused by *Plasmodium berghei* infection and alleviates liver injury by suppressing host’s proinflammatory response. Our study reveals the immune changes in the host of G6pd deficiency during malaria and showed the role of G6pd defect in suppressing the immune response. This study provides new insights into the mechanism of G6pd deficiency mediated resistance against ECM and acute liver injury in the early stage by suppressing the host immune system induced by *P.berghei* parasites and provides an understanding of the malaria natural selection pressure.

## Data Availability Statement

The raw data supporting the conclusions of this article will be made available by the authors, without undue reservation.

## Ethics Statement

The animal study was reviewed and approved by Ethics committee of the Kunming Medical University.

## Author Contributions

YH designed the study. HY, WJ, YL, and WJZ performed the experiments. FY, FL, QL, LL, and WZ analyzed the data. HY and SF wrote the manuscript. YC provided the *Plasmodium berghei* (ANKA 2.34) strain. All authors contributed to the article and approved the submitted version.

## Funding

This work was supported by the National Natural Science Foundation of China (#31760308) and Graduate Innovation Foundation of Kunming Medical University (2021D03).

## Conflict of Interest

The authors declare that the research was conducted in the absence of any commercial or financial relationships that could be construed as a potential conflict of interest.

## Publisher’s Note

All claims expressed in this article are solely those of the authors and do not necessarily represent those of their affiliated organizations, or those of the publisher, the editors and the reviewers. Any product that may be evaluated in this article, or claim that may be made by its manufacturer, is not guaranteed or endorsed by the publisher.
