# Supplementary material for: G6pd-Deficient Mice Are Protected From Experimental Cerebral Malaria and Liver Injury by Suppressing Proinflammatory Response in the Early Stage of Plasmodium berghei Infection
Source: Front Immunol. 2021 Aug 11;12:719189. doi: 10.3389/fimmu.2021.719189 (PMC8386684; doi:10.3389/fimmu.2021.719189)
Supplement: Supplementary file 1 [file DataSheet_1.pdf]

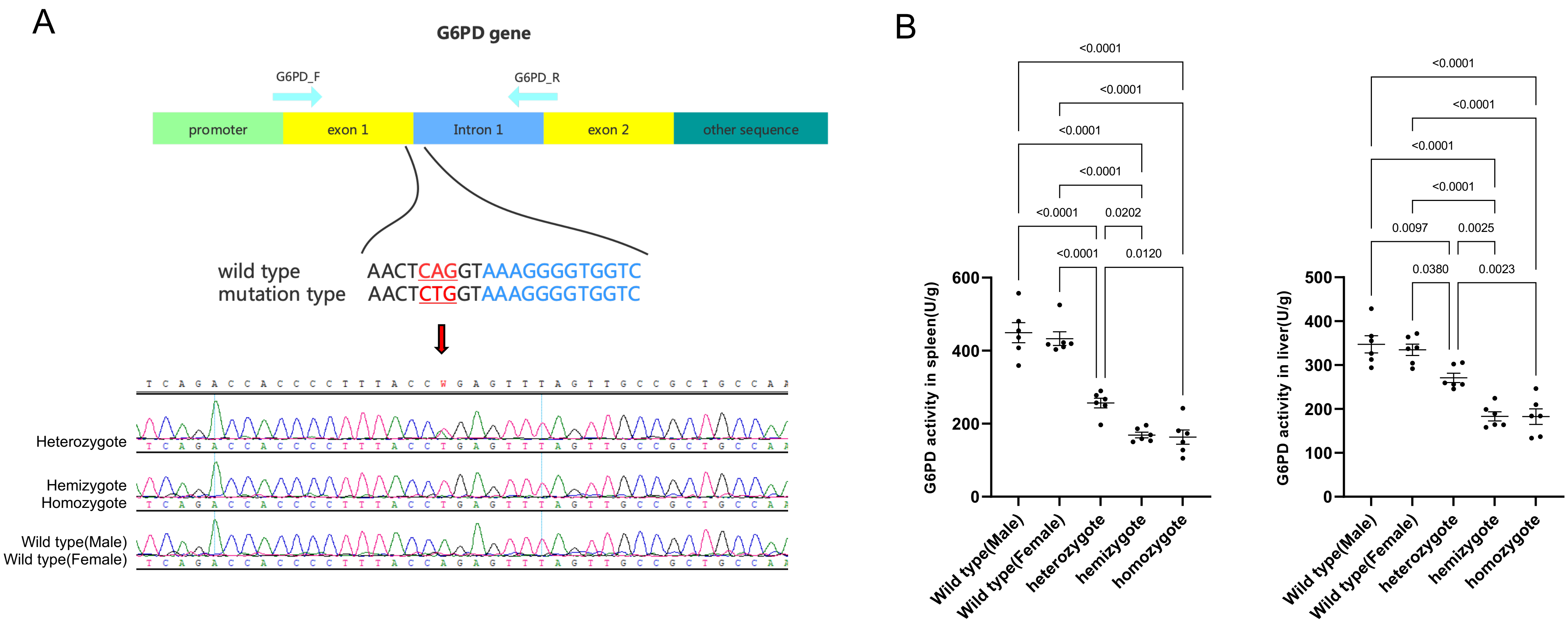

Supplemental Figure.1 G6pd-deficient mice (G6pdx$^{a\text{-mlNeu}}$)

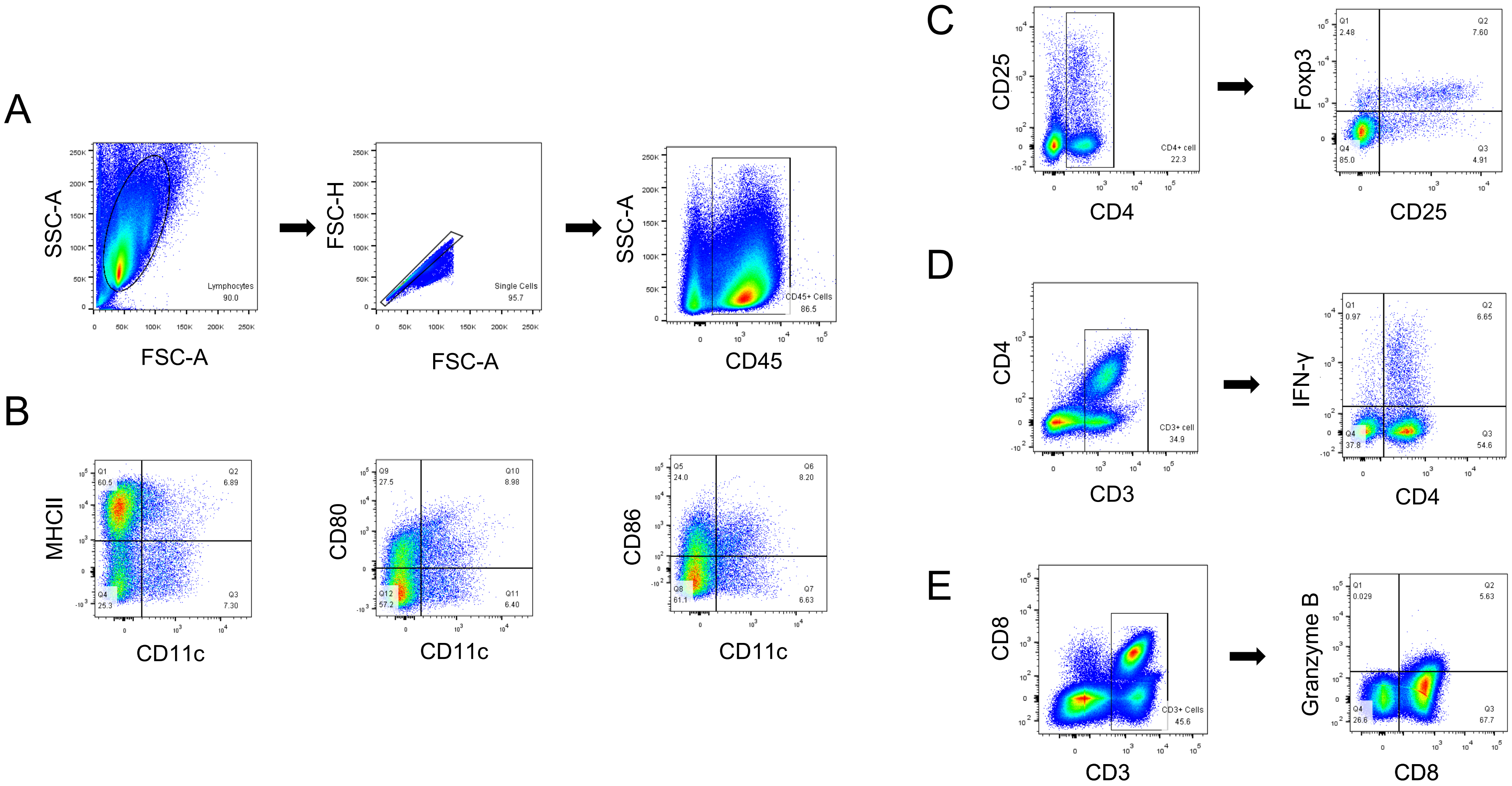

Supplemental Figure.2 Representative gating strategy

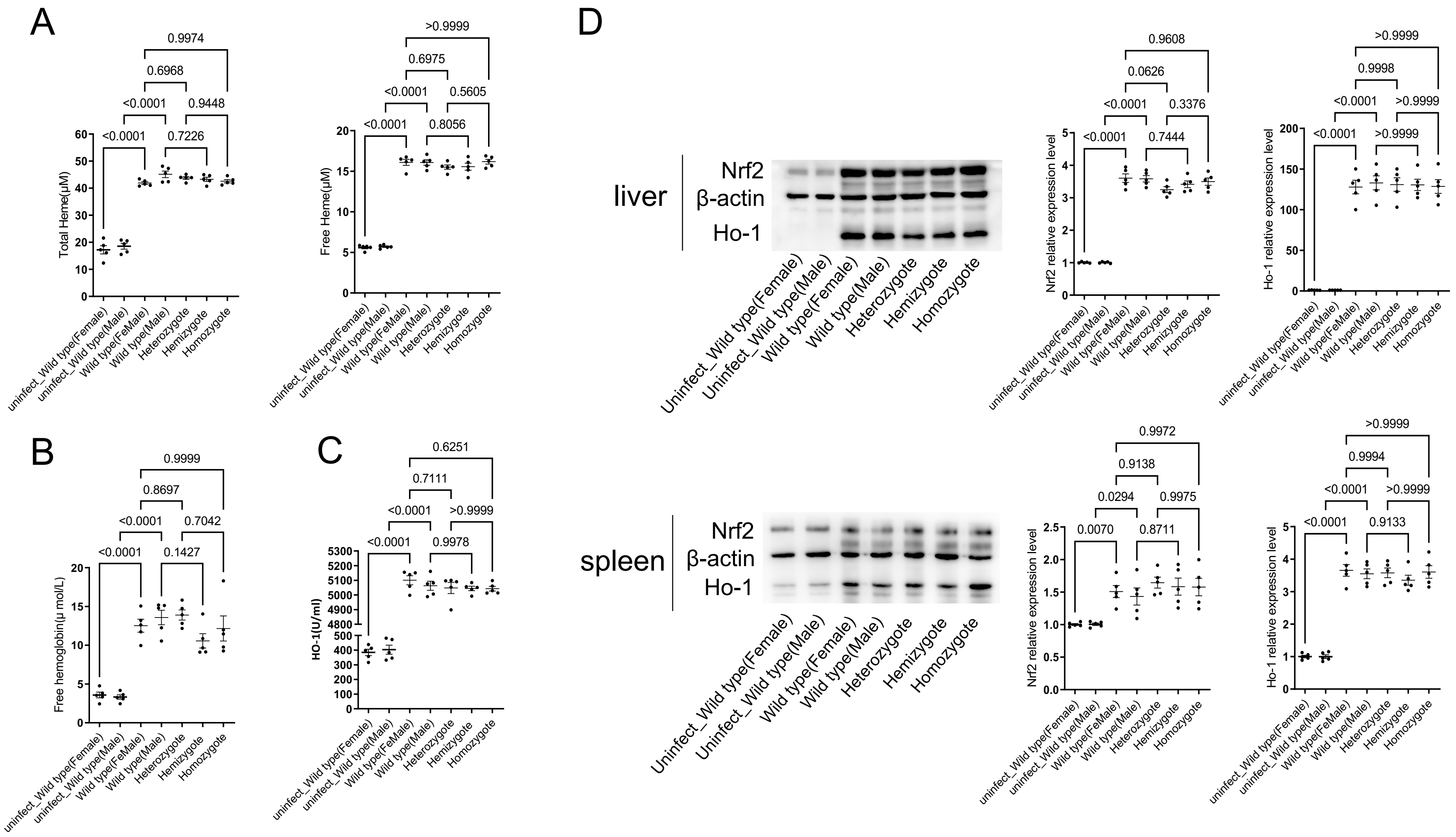

Supplemental Figure.3 G6pd deficiency does not induce hemolysis in the early stages of P. berghei infection.

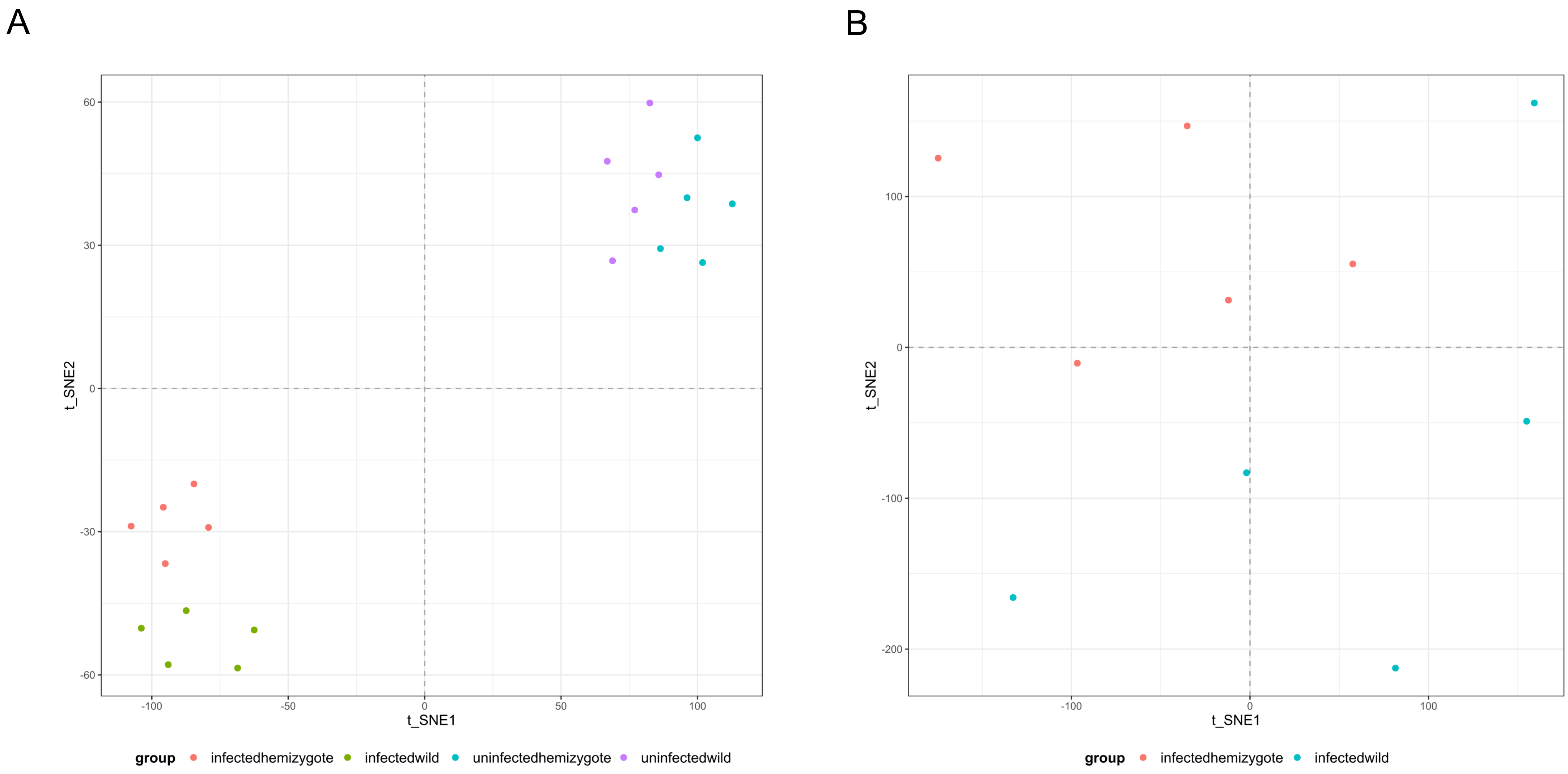

Supplemental Figure.4 tSNE clustering results based on the transcriptome.
