# Supplementary material for: G6pd-Deficient Mice Are Protected From Experimental Cerebral Malaria and Liver Injury by Suppressing Proinflammatory Response in the Early Stage of Plasmodium berghei Infection
Source: Front Immunol. 2021 Aug 11;12:719189. doi: 10.3389/fimmu.2021.719189 (PMC8386684; doi:10.3389/fimmu.2021.719189)
Supplement: Supplementary file 2 [file DataSheet_2.docx]

**Supplemental Table.1 Primer sequence**

| Gene | Primer | Sequence 5’-3’ |
| --- | --- | --- |
| G6pd(PCR) | G6pd_F | GGAAACTGGCTGTGCGCTAC |
|  | G6pd_R (sanger seq) | TCAGCTCCGGCTCTCTTCTG |
| β-actin | β-actin_F | GATTACTGCTCTGGCTCCTAGC |
|  | β-actin_R | GACTCATCGTACTCCTGCTTGC |
| VCAM-1 | VCAM-1_F | CCTCACTTGCAGCACTACGG |
|  | VCAM-1_R | CATGGTCAGAACGGACTTGG |
| ICAM-1 | ICAM-1_F | GGCAGCAAGTAGGCAAGGAC |
|  | ICAM-1_R | CTGGCGGCTCAGTATCTCCT |
| CD36 | CD36_F | CCCTCCAGAATCCAGACAAC |
|  | CD36_R | CACAGGCTTTCCTTCTTTGC |
| CXCL13 | CXCL13_F | GGCCACGGTATTCTGGAAGC |
|  | CXCL13_R | ACCGACAACAGTTGAAATCACTC |
| CXCL2 | CXCL2_F | CATCCAGAGCTTGAGTGTGACG |
|  | CXCL2_R | GGCTTCAGGGTCAAGGCAAACT |
| CCL5 | CCL5_F | TTTGCCTACCTCTCCCTCG |
|  | CCL5_R | CGACTGCAAGATTGGAGCACT |
| CCR1 | CCR1_F | GAACTGGAGCAGAGAGGAAAGA |
|  | CCR1_R | GAAGCACTACAGTGCTCACCA |
| CXCL10 | CXCL10_F | GCCGTCATTTTCTGCCTCAT |
|  | CXCL10_R | GCTTCCCTATGGCCCTCATT |
| CXCR3 | CXCR3_F | AAGTGCCAAAGGCAGAGAAG |
|  | CXCR3_R | AAAGTCCGAGGCATCTAGCA |
| TNF-α | TNF-α_F | TATGGCTCAGGGTCCAACTC |
|  | TNF-α_R | CCCATTTGAGTCCTTGATGG |
| IFN-γ | IFN-γ_F | TGATCCTTTGGACCCTCTGA |
|  | IFN-γ_R | ACAGCCATGAGGAAGAGCTG |
| TGF-β | TGF-β_F | AGCAACAATTCCTGGCGTTACCTT |
|  | TGF-β_R | CCTGTATTCCGTCTCCTTGGTTCAG |
| IL-1β | IL-1β_F | GCTGCTTCCAAACCTTTGAC |
|  | IL-1β_R | AGCTTCTCCACAGCCACAAT |
| IL-6 | IL-6_F | ACAAAGCCAGAGTCCTTCAGAGAGA |
|  | IL-6_R | TGAATTGGATGGTCTTGGTCCTTAGC |
| IL-10 | IL-10_F | CTATGCTGCCTGCTCTTACTGACTG |
|  | IL-10_R | GAGTCGGTTAGCAGTATGTTGTCCAG |
| IL-12 | IL-12_F | TTTGCTGGTGTCTCCACTCA |
|  | IL-12_R | CATCTTCTTCAGGCGTGTCA |

**Supplemental Table.2 Blood routine examination for infected mouse**

| Group | Uninfected | |  | Infect | | | | | P value |
| --- | --- | --- | --- | --- | --- | --- | --- | --- | --- |
|  | Wild type (Female) | Wild type (Male) |  | Wild type (Female) | Wild type (Male) | Heterozygote | Hemizygote | Homozygote |  |
| RBC | 9.44±0.32 | 10.01±0.29 |  | 9.58±0.19 | 9.41±0.24 | 9.58±0.21 | 9.98±0.16 | 10.00±0.45 | 0.5824 |
| HGB | 152.59±5.72 | 150.64±2.86 |  | 148.10±2.13 | 148.00±2.58 | 147.9±3.28 | 146.9±3.09 | 148.2±1.35 | 0.9042 |
| MCV (fL) | 47.78±0.47 | 47.46±0.49 |  | 45.61±0.82 | 47.16±1.02 | 47.76±0.41 | 46.64±1.07 | 45.69±0.52 | 0.3973 |
| MCH (pg) | 16.38±0.33 | 16.08±0.57 |  | 15.47±0.14 | 15.74±0.16 | 15.44±0.05 | 14.72±0.15 | 14.94±0.62 | 0.1159 |
| MCHC(g/L) | 363.72±6.96 | 330.83±6.08 |  | 339.60±7.26 | 334.16±4.96 | 330.23±2.44 | 316.12±5.16 | 326.53±10.76 | 0.1150 |

**Supplemental Table.3 Go enrichment of differentially expressed genes**

|  | GO ID | GO Description | P value | Adjust P value |
| --- | --- | --- | --- | --- |
| *P.berghei* infected wild group compared to the control group | 2376 | immune system process | 1.86E-123 | <1.00E-100 |
|  | 6955 | immune response | 3.47E-83 | 6.76E-80 |
|  | 65007 | biological regulation | 1.96E-75 | 2.60E-72 |
|  | 50896 | response to stimulus | 1.83E-74 | 1.82E-71 |
|  | 9987 | cellular process | 3.85E-72 | 3.06E-69 |
|  | 2682 | regulation of immune system process | 7.30E-69 | 4.83E-66 |
|  | 6952 | defense response | 6.83E-68 | 3.88E-65 |
|  | 50789 | regulation of biological process | 2.55E-67 | 1.27E-64 |
|  | 48518 | positive regulation of biological process | 1.06E-65 | 4.71E-63 |
|  | 48583 | regulation of response to stimulus | 1.41E-59 | 5.63E-57 |
|  | 2684 | positive regulation of immune system process | 4.75E-59 | 1.60E-56 |
|  | 1775 | cell activation | 4.89E-59 | 1.60E-56 |
|  | 6950 | response to stress | 1.666E-58 | 5.09E-56 |
|  | 50794 | regulation of cellular process | 4.9126E-56 | 1.39E-53 |
|  | 45321 | leukocyte activation | 8.5628E-55 | 2.26E-52 |
|  | 50776 | regulation of immune response | 6.8255E-54 | 1.69E-51 |
|  | 9611 | response to wounding | 9.8571E-51 | 2.30E-48 |
|  | 48522 | positive regulation of cellular process | 2.5881E-49 | 5.71E-47 |
|  | 6954 | inflammatory response | 8.8217E-49 | 1.84E-46 |
| *P.berghei* infected hemizygote group compared to the *P.berghei* infected wild group | 50896 | response to stimulus | 6.07E-17 | 1.78E-13 |
|  | 6950 | response to stress | 1.48E-12 | 2.18E-09 |
|  | 5488 | binding | 1.31E-11 | 1.28E-08 |
|  | 9611 | response to wounding | 4.53E-11 | 3.33E-08 |
|  | 9987 | cellular process | 1.03E-10 | 6.03E-08 |
|  | 5576 | extracellular region | 1.70E-10 | 8.30E-08 |
|  | 6954 | inflammatory response | 2.55E-10 | 1.07E-07 |
|  | 44421 | extracellular region part | 1.05E-09 | 3.85E-07 |
|  | 6952 | defense response | 1.78E-09 | 5.80E-07 |
|  | 42221 | response to chemical stimulus | 8.83E-09 | 2.59E-06 |
|  | 6955 | immune response | 1.49E-08 | 3.97E-06 |
|  | 5615 | extracellular space | 2.04E-08 | 5.00E-06 |
|  | 2687 | positive regulation of leukocyte migration | 4.00E-08 | 9.03E-06 |
|  | 44464 | cell part | 6.07E-08 | 1.14E-05 |
|  | 5623 | cell | 6.12E-08 | 1.14E-05 |
|  | 3824 | catalytic activity | 6.22E-08 | 1.14E-05 |
|  | 4497 | monooxygenase activity | 1.08E-07 | 1.86E-05 |
|  | 5886 | plasma membrane | 1.15E-07 | 1.87E-05 |
|  | 16020 | membrane | 1.82E-07 | 2.81E-05 |
|  | 2685 | regulation of leukocyte migration | 2.56E-07 | 3.76E-05 |
